# Crystal structure of bis­(3-bromo­pyridine-κ*N*)bis­(*O*-ethyl di­thio­carbonato-κ^2^ *S*,*S*′)nickel(II)

**DOI:** 10.1107/S2056989014027339

**Published:** 2015-01-01

**Authors:** Rajni Kant, Gurvinder Kour, Sumati Anthal, Neerupama, Renu Sachar

**Affiliations:** a Post-Graduate Department of Physics & Electronics, University of Jammu, Jammu Tawi 180 006, India; b Post-Graduate Department of Chemistry, University of Jammu, Jammu Tawi 180 006, India

**Keywords:** Crystal structure, nickel complex, xanthate ligands, π–π inter­actions, crystal structure

## Abstract

In the title mol­ecular complex, [Ni(C_3_H_5_OS_2_)_2_(C_5_H_4_BrN)_2_], the Ni^2+^ cation is located on a centre of inversion and has a distorted octa­hedral N_2_S_4_ environment defined by two chelating xanthate ligands and two monodentate pyridine ligands. The C—S bond lengths of the thio­carboxyl­ate group are indicative of a delocalized bond and the O—C*sp* ^2^ bond is considerably shorter than the O—C*sp* ^3^ bond, consistent with a significant contribution of one resonance form of the xanthate anion that features a formal C=O_+_ unit and a negative charge on each of the S atoms. The packing of the mol­ecules is stabilized by C—H⋯S and C—H⋯π inter­actions. In addition, π–π inter­actions between the pyridine rings [centroid-to-centroid distance = 3.797 (3) Å] are also present. In the crystal structure, mol­ecules are arranged in rows along [100], forming layers parallel to (010) and (001).

## Related literature   

Xanthates as ligands have been investigated extensively due to their coordination behaviour (Haiduc *et al.*, 1995 ▸), thereby showing monodentate and/or bidentate coordination modes (Xiong *et al.*, 1997 ▸; Trávnícek *et al.*, 1995 ▸). Xanthates have also found uses as anti­tumour agents and in the treatment of Alzheimer’s disease (Orts *et al.*, 2002 ▸; Larsson & Öberg, 2011 ▸). For other analogous Ni–di­thio­carboxyl­ate complexes, see: Kapoor *et al.* (2012 ▸). For C—S and C—O bond lengths in other xanthates, see: Jiang *et al.* (2002 ▸); Alam *et al.* (2011 ▸).
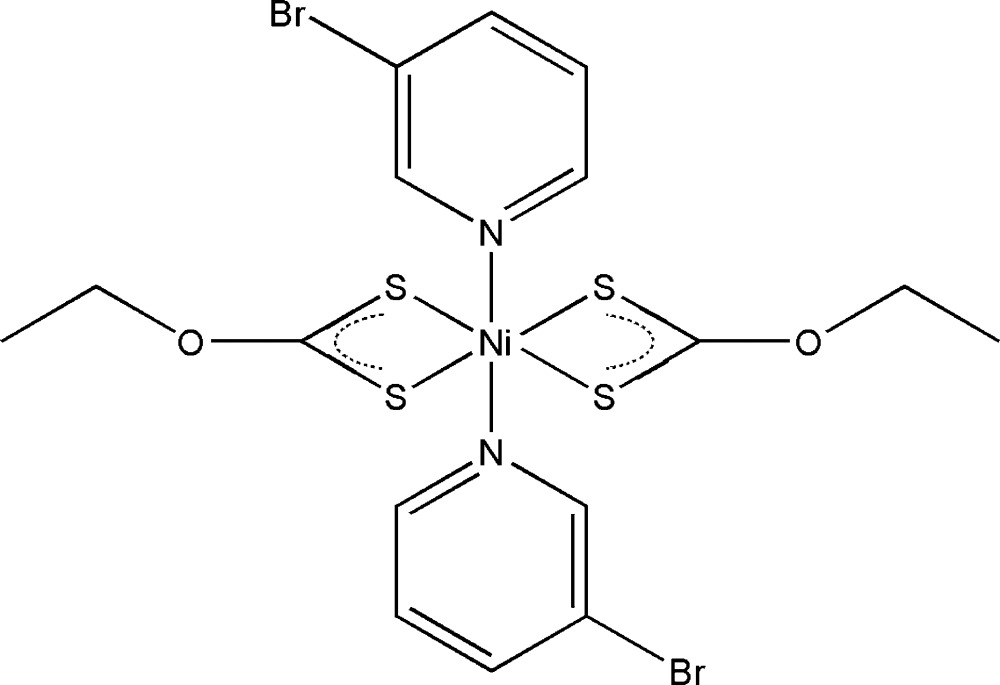


## Experimental   

### Crystal data  

[Ni(C_3_H_5_OS_2_)_2_(C_5_H_4_BrN)_2_]
*M* *_r_* = 617.09
Triclinic, 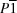

*a* = 6.8397 (7) Å
*b* = 9.1952 (8) Å
*c* = 9.7562 (10) Å
α = 76.121 (8)°
β = 73.935 (9)°
γ = 78.517 (8)°
*V* = 566.59 (10) Å^3^
*Z* = 1
Mo *K*α radiation
μ = 4.77 mm^−1^
*T* = 293 K
0.3 × 0.2 × 0.1 mm

### Data collection  

Oxford Diffraction Xcalibur CCD diffractometer
Absorption correction: multi-scan (*CrysAlis RED*; Oxford Diffraction, 2010 ▸) *T*_min_ = 0.489, *T*_max_ = 1.000
4016 measured reflections
2230 independent reflections
1510 reflections with *I* > 2σ(*I*)
*R*_int_ = 0.044

### Refinement  

*R*[*F* ^2^ > 2σ(*F* ^2^)] = 0.049
*wR*(*F* ^2^) = 0.112
*S* = 1.03
2230 reflections
126 parameters
H-atom parameters constrained
Δρ_max_ = 0.67 e Å^−3^
Δρ_min_ = −0.68 e Å^−3^

### 

Data collection: *CrysAlis PRO* (Oxford Diffraction, 2010 ▸); cell refinement: *CrysAlis PRO*; data reduction: *CrysAlis PRO*; program(s) used to solve structure: *SHELXS97* (Sheldrick, 2008 ▸); program(s) used to refine structure: *SHELXL97* (Sheldrick, 2008 ▸); molecular graphics: *ORTEP-3 for Windows* (Farrugia, 2012 ▸); software used to prepare material for publication: *PLATON* (Spek, 2009 ▸) and *PARST* (Nardelli, 1995 ▸).

## Supplementary Material

Crystal structure: contains datablock(s) I, New_Global_Publ_Block. DOI: 10.1107/S2056989014027339/wm5101sup1.cif

Structure factors: contains datablock(s) I. DOI: 10.1107/S2056989014027339/wm5101Isup2.hkl

**Fig. 1.**
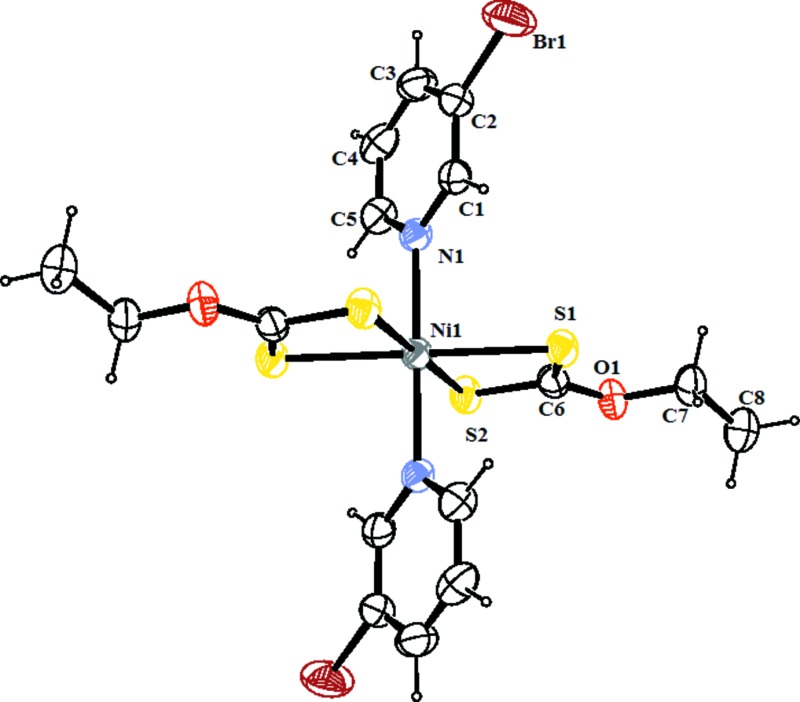
The molecular structure of the title compound, with the atom-labelling scheme. Displacement ellipsoids are drawn at the 40% probability level. H atoms are shown as small spheres of arbitrary radius. All non-labelled atoms are related by symmetry code (-x+1, -y, -z).

. DOI: 10.1107/S2056989014027339/wm5101fig1.tif
The mol­ecular structure of the title compound, with the atom-labelling scheme. Displacement ellipsoids are drawn at the 40% probability level. H atoms are shown as small spheres of arbitrary radius. All non-labelled atoms are related by symmetry code (-x+1, −y, −z).

**Fig. 2.**
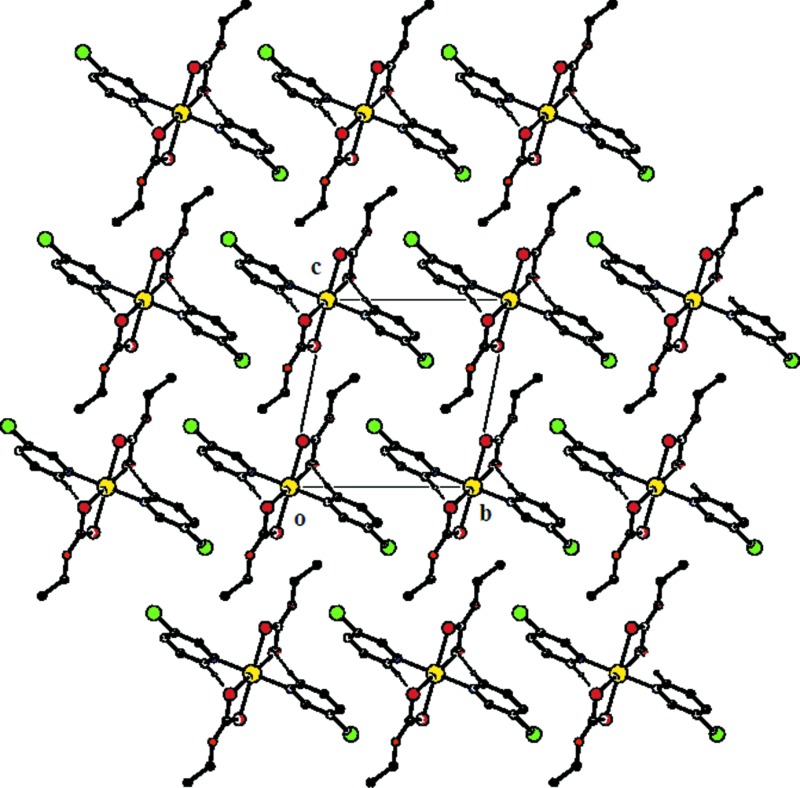
The packing arrangement of molecules of the title compound viewed down [100].

. DOI: 10.1107/S2056989014027339/wm5101fig2.tif
The packing arrangement of mol­ecules of the title compound viewed down [100].

CCDC reference: 1036070

Additional supporting information:  crystallographic information; 3D view; checkCIF report

## Figures and Tables

**Table 1:** Selected bond lengths ()

Ni1N1	2.118(4)
Ni1S2	2.4314(12)
Ni1S1	2.4368(12)
S2C6	1.691(5)
S1C6	1.679(5)
C6O1	1.328(5)
C7O1	1.447(5)

**Table 2:** Hydrogen-bond geometry (, ) *Cg*1 is the centroid of the N1/C1/C2/C3/C4/C5 ring.

*D*H*A*	*D*H	H*A*	*D* *A*	*D*H*A*
C5H5S2^i^	0.93	2.78	3.642(5)	154
C8H8*A* *Cg*1^ii^	0.96	3.26	3.712(6)	111

Symmetry codes: (i) 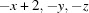; (ii) 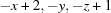.

## supplementary crystallographic information

### S1. Experimental

Bis(*O*-ethyldithiocarbonato)nickel(II) required for preparation of the adduct was obtained by mixing aqueous solutions of the potassium salt of *O*-ethyldithiocarbonate (3.24 g, 0.02 mol) and NiCl_2_·6H_2_O (2.37 g, 0.01 mol). The formed bis(*O*-ethyldithiocarbonato)nickel(II) precipitate was immediately filtered off and dried in a vacuum desiccator. Bis(*O*-ethyldithiocarbonato)nickel(II) (0.783 g, 0.0026 mol) was then dissolved in acetone (60 ml) and stirred for about 10–20 minutes. To the resulting solution, 3-bromopyridine (0.82 g, 0.0052 mol) was added. The mixture was stirred for additional two to three hours and kept undisturbed for one to two days when dark green coloured crystals of the adduct had formed. The product so obtained was filtered and dried in vacuum desiccator over anhydrous calcium chloride.

### S2. Refinement

All H atoms were positioned geometrically and refined using a riding model, with C—H = 0.93–0.97 Å, with *U*_iso_(H) = 1.2*U*_eq_(C), except for the methyl group where *U*_iso_(H) = 1.5*U*_eq_(C).

### Crystal data

**Table:**

[Ni(C_3_H_5_OS_2_)_2_(C_5_H_4_BrN)_2_]	*Z* = 1
*M**_r_* = 617.09	*F*(000) = 306
Triclinic, *P*1	*D*_x_ = 1.809 Mg m^−^^3^
Hall symbol: -P 1	Mo *K*α radiation, λ = 0.71073 Å
*a* = 6.8397 (7) Å	Cell parameters from 1227 reflections
*b* = 9.1952 (8) Å	θ = 4.1–27.4°
*c* = 9.7562 (10) Å	µ = 4.77 mm^−^^1^
α = 76.121 (8)°	*T* = 293 K
β = 73.935 (9)°	Block, dark green
γ = 78.517 (8)°	0.3 × 0.2 × 0.1 mm
*V* = 566.59 (10) Å^3^	

### Data collection

**Table:**

Oxford Diffraction Xcalibur CCD diffractometer	2230 independent reflections
Radiation source: fine-focus sealed tube	1510 reflections with *I* > 2σ(*I*)
Graphite monochromator	*R*_int_ = 0.044
ω scans	θ_max_ = 26.0°, θ_min_ = 3.6°
Absorption correction: multi-scan (*CrysAlis RED*; Oxford Diffraction, 2010)	*h* = −6→8
*T*_min_ = 0.489, *T*_max_ = 1.000	*k* = −9→11
4016 measured reflections	*l* = −11→12

### Refinement

**Table:**

Refinement on *F*^2^	Secondary atom site location: difference Fourier map
Least-squares matrix: full	Hydrogen site location: inferred from neighbouring sites
*R*[*F*^2^ > 2σ(*F*^2^)] = 0.049	H-atom parameters constrained
*wR*(*F*^2^) = 0.112	*w* = 1/[σ^2^(*F*_o_^2^) + (0.0339*P*)^2^] where *P* = (*F*_o_^2^ + 2*F*_c_^2^)/3
*S* = 1.03	(Δ/σ)_max_ = 0.001
2230 reflections	Δρ_max_ = 0.67 e Å^−^^3^
126 parameters	Δρ_min_ = −0.68 e Å^−^^3^
0 restraints	Extinction correction: *SHELXL97* (Sheldrick, 2008), Fc^*^=kFc[1+0.001xFc^2^λ^3^/sin(2θ)]^-1/4^
Primary atom site location: structure-invariant direct methods	Extinction coefficient: 0.0130 (18)

### Special details

**Table:**

Experimental. *CrysAlis PRO*, Agilent Technologies, Version 1.171.36.28 (release 01–02-2013 CrysAlis171. NET) (compiled Feb 1 2013,16:14:44) Empirical absorption correction using spherical harmonics, implemented in SCALE3 ABSPACK scaling algorithm.
Geometry. All e.s.d.'s (except the e.s.d. in the dihedral angle between two l.s. planes) are estimated using the full covariance matrix. The cell e.s.d.'s are taken into account individually in the estimation of e.s.d.'s in distances, angles and torsion angles; correlations between e.s.d.'s in cell parameters are only used when they are defined by crystal symmetry. An approximate (isotropic) treatment of cell e.s.d.'s is used for estimating e.s.d.'s involving l.s. planes.
Refinement. Refinement of *F*^2^ against ALL reflections. The weighted *R*-factor *wR* and goodness of fit *S* are based on *F*^2^, conventional *R*-factors *R* are based on *F*, with *F* set to zero for negative *F*^2^. The threshold expression of *F*^2^ > σ(*F*^2^) is used only for calculating *R*-factors(gt) *etc*. and is not relevant to the choice of reflections for refinement. *R*-factors based on *F*^2^ are statistically about twice as large as those based on *F*, and *R*- factors based on ALL data will be even larger.

### Fractional atomic coordinates and isotropic or equivalent isotropic displacement parameters (Å^2^)

**Table:**

	*x*	*y*	*z*	*U*_iso_*/*U*_eq_	
Ni1	0.5000	0.0000	0.0000	0.0371 (3)	
Br1	0.31297 (11)	0.60432 (7)	−0.32487 (8)	0.0877 (3)	
S2	0.85033 (18)	−0.09955 (15)	−0.11048 (14)	0.0456 (4)	
S1	0.49994 (18)	−0.02146 (15)	−0.24413 (14)	0.0451 (4)	
C4	0.7696 (8)	0.4151 (6)	−0.1093 (6)	0.0552 (15)	
H4	0.8821	0.4427	−0.0908	0.066*	
C6	0.7488 (7)	−0.0893 (5)	−0.2527 (5)	0.0418 (12)	
C7	0.8014 (8)	−0.1290 (6)	−0.4944 (5)	0.0540 (15)	
H7A	0.7966	−0.0258	−0.5491	0.065*	
H7B	0.6644	−0.1570	−0.4674	0.065*	
O1	0.8752 (5)	−0.1419 (4)	−0.3659 (3)	0.0484 (9)	
C8	0.9484 (8)	−0.2339 (7)	−0.5834 (6)	0.0742 (19)	
H8A	1.0845	−0.2083	−0.6050	0.111*	
H8B	0.9089	−0.2248	−0.6724	0.111*	
H8C	0.9463	−0.3361	−0.5299	0.111*	
C1	0.4523 (7)	0.3245 (5)	−0.1639 (5)	0.0450 (13)	
H1	0.3439	0.2931	−0.1841	0.054*	
C5	0.7205 (7)	0.2725 (6)	−0.0545 (6)	0.0474 (13)	
H5	0.7986	0.2055	0.0044	0.057*	
C2	0.4894 (8)	0.4701 (6)	−0.2192 (5)	0.0479 (13)	
C3	0.6516 (8)	0.5174 (6)	−0.1919 (6)	0.0565 (15)	
H3	0.6801	0.6161	−0.2285	0.068*	
N1	0.5662 (5)	0.2245 (4)	−0.0814 (4)	0.0392 (10)	

### Atomic displacement parameters (Å^2^)

**Table:**

	*U*^11^	*U*^22^	*U*^33^	*U*^12^	*U*^13^	*U*^23^
Ni1	0.0384 (5)	0.0377 (5)	0.0395 (6)	0.0025 (4)	−0.0206 (4)	−0.0089 (4)
Br1	0.1128 (6)	0.0498 (4)	0.1009 (6)	−0.0010 (4)	−0.0560 (5)	0.0109 (4)
S2	0.0407 (7)	0.0547 (9)	0.0446 (8)	0.0053 (6)	−0.0205 (6)	−0.0137 (6)
S1	0.0465 (7)	0.0503 (8)	0.0439 (8)	0.0035 (7)	−0.0243 (6)	−0.0124 (6)
C4	0.048 (3)	0.057 (4)	0.065 (4)	−0.015 (3)	−0.002 (3)	−0.028 (3)
C6	0.046 (3)	0.042 (3)	0.034 (3)	−0.002 (2)	−0.008 (2)	−0.006 (2)
C7	0.061 (3)	0.064 (4)	0.037 (3)	−0.009 (3)	−0.008 (3)	−0.016 (3)
O1	0.0487 (19)	0.065 (2)	0.033 (2)	−0.0033 (18)	−0.0124 (16)	−0.0125 (18)
C8	0.097 (5)	0.077 (5)	0.049 (4)	−0.008 (4)	−0.010 (3)	−0.026 (3)
C1	0.053 (3)	0.043 (3)	0.044 (3)	−0.002 (3)	−0.019 (2)	−0.011 (3)
C5	0.041 (3)	0.051 (3)	0.054 (3)	−0.004 (3)	−0.015 (2)	−0.015 (3)
C2	0.059 (3)	0.040 (3)	0.041 (3)	−0.006 (3)	−0.009 (2)	−0.006 (2)
C3	0.068 (4)	0.040 (3)	0.061 (4)	−0.014 (3)	−0.009 (3)	−0.010 (3)
N1	0.040 (2)	0.039 (2)	0.043 (3)	−0.0020 (19)	−0.0167 (19)	−0.0109 (19)

### Geometric parameters (Å, º)

**Table:**

Ni1—N1^i^	2.118 (4)	C7—C8	1.492 (6)
Ni1—N1	2.118 (4)	C7—H7A	0.9700
Ni1—S2^i^	2.4314 (12)	C7—H7B	0.9700
Ni1—S2	2.4314 (12)	C8—H8A	0.9600
Ni1—S1	2.4368 (12)	C8—H8B	0.9600
Ni1—S1^i^	2.4368 (12)	C8—H8C	0.9600
Br1—C2	1.878 (5)	C1—N1	1.344 (5)
S2—C6	1.691 (5)	C1—C2	1.364 (7)
S1—C6	1.679 (5)	C1—H1	0.9300
C4—C5	1.363 (7)	C5—N1	1.331 (6)
C4—C3	1.372 (8)	C5—H5	0.9300
C4—H4	0.9300	C2—C3	1.379 (7)
C6—O1	1.328 (5)	C3—H3	0.9300
C7—O1	1.447 (5)		
			
N1^i^—Ni1—N1	180.0 (3)	O1—C7—H7B	110.4
N1^i^—Ni1—S2^i^	90.75 (10)	C8—C7—H7B	110.4
N1—Ni1—S2^i^	89.25 (10)	H7A—C7—H7B	108.6
N1^i^—Ni1—S2	89.25 (10)	C6—O1—C7	118.9 (3)
N1—Ni1—S2	90.75 (10)	C7—C8—H8A	109.5
S2^i^—Ni1—S2	180.00 (8)	C7—C8—H8B	109.5
N1^i^—Ni1—S1	90.29 (11)	H8A—C8—H8B	109.5
N1—Ni1—S1	89.71 (11)	C7—C8—H8C	109.5
S2^i^—Ni1—S1	106.15 (4)	H8A—C8—H8C	109.5
S2—Ni1—S1	73.85 (4)	H8B—C8—H8C	109.5
N1^i^—Ni1—S1^i^	89.71 (11)	N1—C1—C2	122.6 (5)
N1—Ni1—S1^i^	90.29 (11)	N1—C1—H1	118.7
S2^i^—Ni1—S1^i^	73.85 (4)	C2—C1—H1	118.7
S2—Ni1—S1^i^	106.15 (4)	N1—C5—C4	123.2 (5)
S1—Ni1—S1^i^	180.000 (5)	N1—C5—H5	118.4
C6—S2—Ni1	82.79 (15)	C4—C5—H5	118.4
C6—S1—Ni1	82.87 (17)	C1—C2—C3	119.3 (5)
C5—C4—C3	119.3 (5)	C1—C2—Br1	119.4 (4)
C5—C4—H4	120.3	C3—C2—Br1	121.2 (4)
C3—C4—H4	120.3	C4—C3—C2	118.2 (5)
O1—C6—S1	123.4 (3)	C4—C3—H3	120.9
O1—C6—S2	116.1 (3)	C2—C3—H3	120.9
S1—C6—S2	120.5 (3)	C5—N1—C1	117.4 (5)
O1—C7—C8	106.8 (4)	C5—N1—Ni1	122.2 (3)
O1—C7—H7A	110.4	C1—N1—Ni1	120.5 (3)
C8—C7—H7A	110.4		
			
N1^i^—Ni1—S2—C6	89.6 (2)	N1—C1—C2—Br1	−176.7 (3)
N1—Ni1—S2—C6	−90.4 (2)	C5—C4—C3—C2	−1.5 (8)
S1—Ni1—S2—C6	−0.95 (18)	C1—C2—C3—C4	−0.1 (7)
S1^i^—Ni1—S2—C6	179.05 (18)	Br1—C2—C3—C4	177.5 (4)
N1^i^—Ni1—S1—C6	−88.2 (2)	C4—C5—N1—C1	−1.6 (7)
N1—Ni1—S1—C6	91.8 (2)	C4—C5—N1—Ni1	178.0 (4)
S2^i^—Ni1—S1—C6	−179.04 (18)	C2—C1—N1—C5	−0.1 (7)
S2—Ni1—S1—C6	0.96 (18)	C2—C1—N1—Ni1	−179.8 (3)
Ni1—S1—C6—O1	176.2 (4)	S2^i^—Ni1—N1—C5	125.0 (3)
Ni1—S1—C6—S2	−1.5 (3)	S2—Ni1—N1—C5	−55.0 (3)
Ni1—S2—C6—O1	−176.4 (4)	S1—Ni1—N1—C5	−128.9 (3)
Ni1—S2—C6—S1	1.5 (3)	S1^i^—Ni1—N1—C5	51.1 (3)
S1—C6—O1—C7	5.5 (6)	S2^i^—Ni1—N1—C1	−55.4 (3)
S2—C6—O1—C7	−176.7 (4)	S2—Ni1—N1—C1	124.6 (3)
C8—C7—O1—C6	−164.0 (5)	S1—Ni1—N1—C1	50.8 (3)
C3—C4—C5—N1	2.5 (8)	S1^i^—Ni1—N1—C1	−129.2 (3)
N1—C1—C2—C3	1.0 (7)		

Symmetry code: (i) −*x*+1, −*y*, −*z*.

### Hydrogen-bond geometry (Å, º)

Cg1 is the centroid of the N1/C1/C2/C3/C4/C5 ring.

**Table:**

*D*—H···*A*	*D*—H	H···*A*	*D*···*A*	*D*—H···*A*
C5—H5···S2^ii^	0.93	2.78	3.642 (5)	154
C8—H8*A*···*Cg*1^iii^	0.96	3.26	3.712 (6)	111

Symmetry codes: (ii) −*x*+2, −*y*, −*z*; (iii) −*x*+2, −*y*, −*z*+1.

## References

[bb1] Alam, N., Ehsan, M. A., Zeller, M., Mazhar, M. & Arifin, Z. (2011). *Acta Cryst.* E**67**, m1064. 10.1107/S1600536811026523 PMC3212145 22090847

[bb2] Farrugia, L. J. (2012). *J. Appl. Cryst.* **45**, 849–854.

[bb3] Haiduc, I., Sowerby, D. B. & Lu, S. F. (1995). *Polyhedron*, **14**, 3389–3472.

[bb4] Jiang, X. H., Zhang, W. G., Zhong, Y. & Wang, S. L. (2002). *Molecules*, **7**, 549–553.

[bb5] Kapoor, S., Sachar, R., Singh, K., Gupta, V. K. & Rajnikant, V. (2012). *J. Chem. Crystallogr.* **42**, 222–226.

[bb6] Larsson, A. C. & Öberg, S. (2011). *J. Phys. Chem. A*, **115**, 1396–1407. 10.1021/jp110233d 21309541

[bb7] Nardelli, M. (1995). *J. Appl. Cryst.* **28**, 659.

[bb8] Orts, W. J., Sojka, R. E. & Glenn, G. M. (2002). *Agro Food Ind.* **13**, 37–41.

[bb9] Oxford Diffraction (2010). *CrysAlis RED* and *CrysAlis PRO*. Oxford Diffraction Ltd, Yarnton, England.

[bb10] Sheldrick, G. M. (2008). *Acta Cryst.* A**64**, 112–122. 10.1107/S0108767307043930 18156677

[bb11] Spek, A. L. (2009). *Acta Cryst.* D**65**, 148–155. 10.1107/S090744490804362X PMC2631630 19171970

[bb12] Trávnícek, Z., Pastorek, R., Sindelár, Z., Klicka, R. & Marek, J. (1995). *Polyhedron*, **14**, 3627–3633.

[bb13] Xiong, R.-G., Zh, Y., Liu, C.-M. & You, X.-Z. (1997). *Polyhedron*, **16**, 2667–2671.

