# Supplementary figures and images for: Crystal structure of bis­(3-bromo­pyridine-κN)bis­(O-ethyl di­thio­carbonato-κ2 S,S′)nickel(II)
Source: Acta Crystallogr E Crystallogr Commun. 2015 Jan 1;71(Pt 1):m12–3. doi: 10.1107/S2056989014027339 (PMC4331872; doi:10.1107/S2056989014027339)

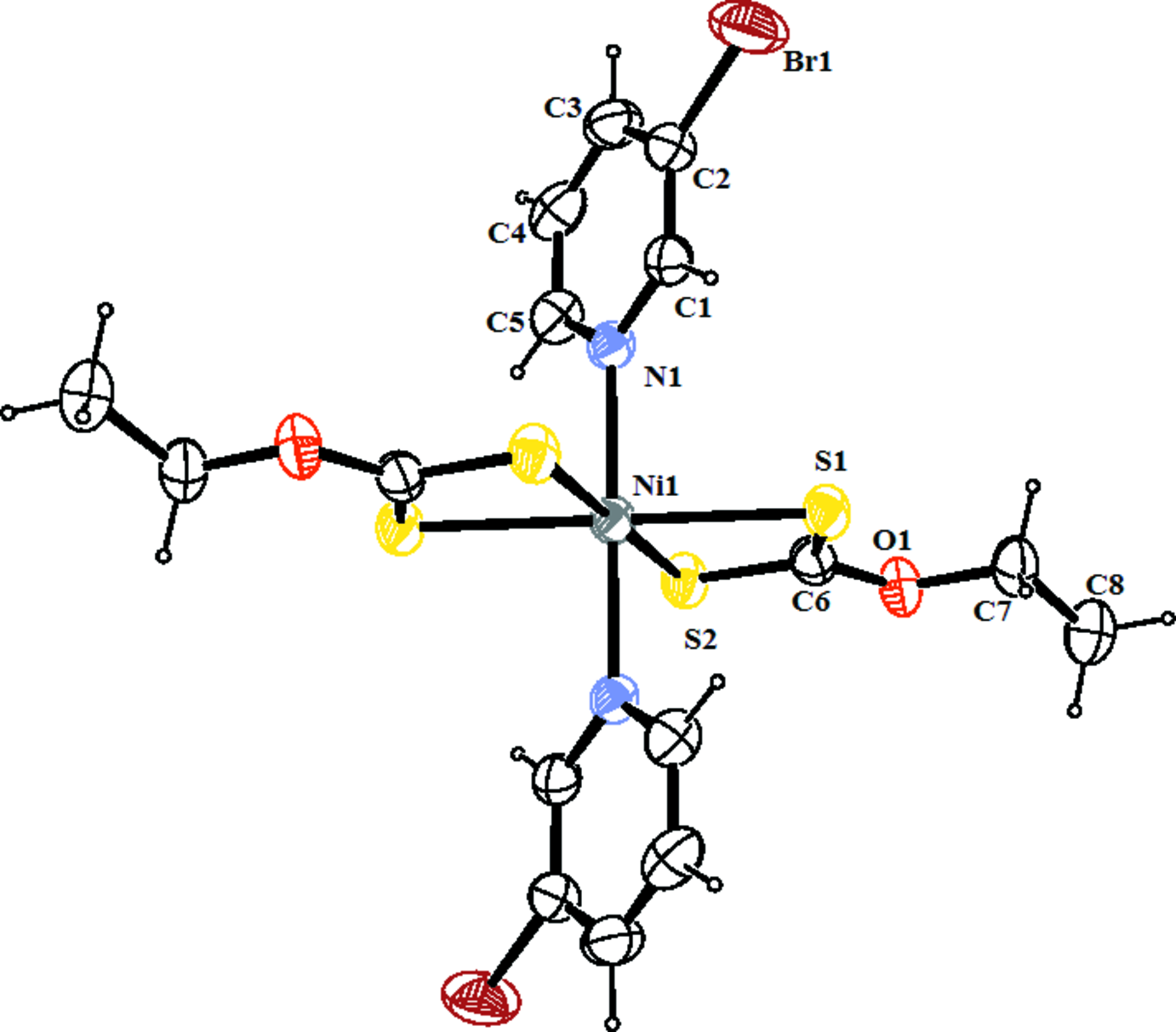

Supplement: Supplementary file 3 [file e-71-00m12-fig1.tif]

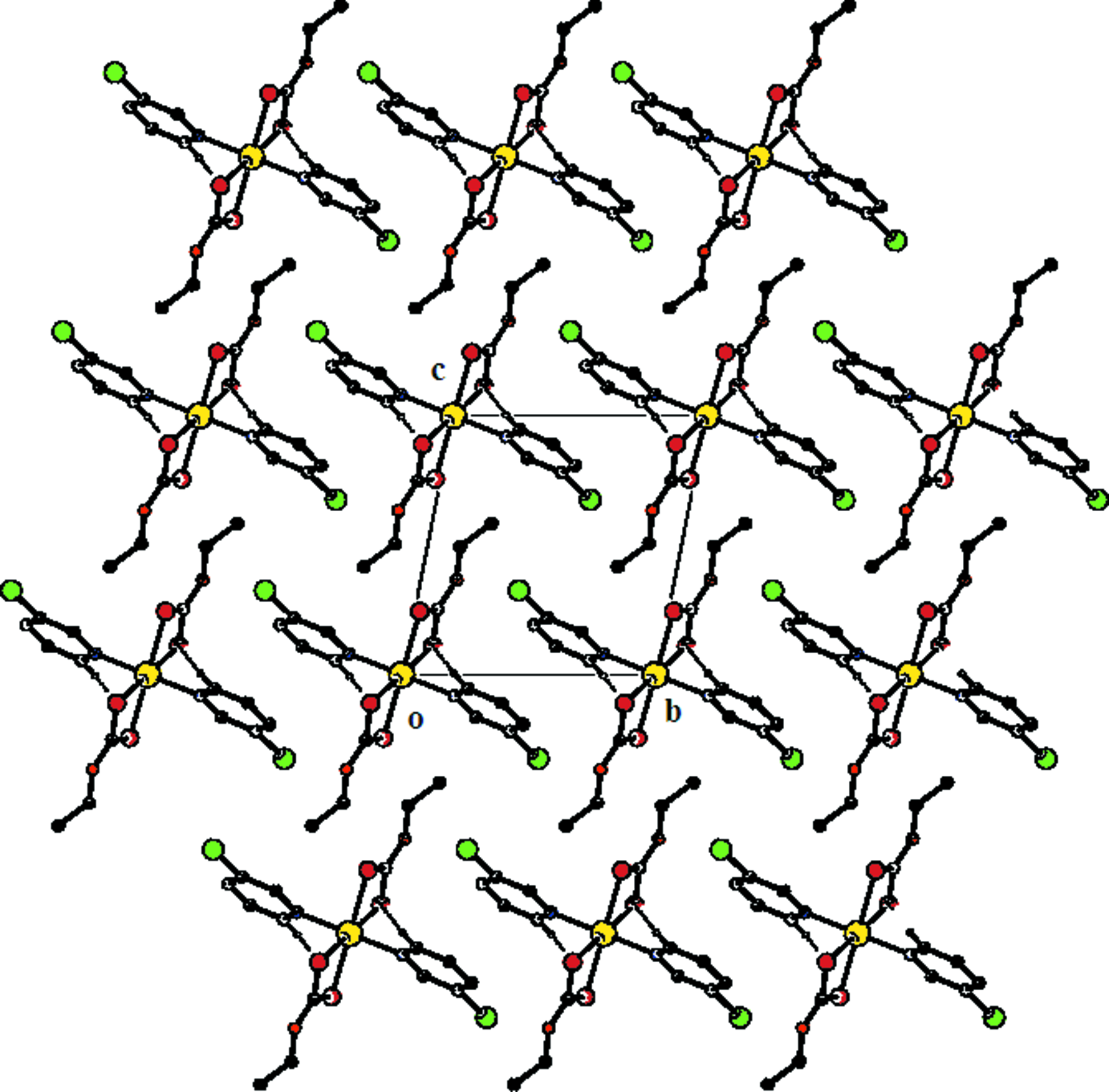

Supplement: Supplementary file 4 [file e-71-00m12-fig2.tif]
